# Adaptive evolution of SARS-CoV-2 during a persistent infection for 521 days in an immunocompromised patient

**DOI:** 10.1038/s41525-025-00463-x

**Published:** 2025-01-17

**Authors:** Hanno Schmidt, Lea Schick, Jürgen Podlech, Angélique Renzaho, Bettina Lieb, Stefan Diederich, Thomas Hankeln, Bodo Plachter, Oliver Kriege

**Affiliations:** 1 Sequencing Consortium, University Medical Center of the Johannes Gutenberg-University Mainz, Mainz, Germany; 2 Institute of Virology, University Medical Center of the Johannes Gutenberg-University Mainz, Mainz, Germany; 3 Institute for Quantitative and Computational Biosciences, Johannes Gutenberg-University Mainz, Mainz, Germany; 4 Third Department of Medicine—Hematology, Internal Oncology, and Pneumology, University Medical Center of the Johannes Gutenberg-University Mainz, Mainz, Germany; 5 StarSEQ GmbH, Mainz, Germany; 6 Institute of Human Genetics, University Medical Center of the Johannes Gutenberg-University Mainz, Mainz, Germany; 7 Institute of Organismal and Molecular Evolutionary Biology, Johannes Gutenberg-University Mainz, Mainz, Germany

**Keywords:** Genetics, Diseases

## Abstract

Immunocompromised patients struggle to adequately clear viral infections, offering the virus the opportunity to adapt to the immune system in the host. Here we present a case study of a patient undergoing allogeneic hematopoietic stem cell transplantation with a 521-day follow-up of a SARS-CoV-2 infection with the BF.7.21 variant. Virus samples from five time points were submitted to whole genome sequencing. Between the first detection of SARS-CoV-2 infection and its clearance, the patient’s virus population acquired 34 amino acid substitutions and 8 deletions in coding regions. With 11 amino acid substitutions in the receptor binding domain of the virus’ spike protein, substitutions were 15 times more abundant than expected for a random distribution in this highly functional region. Amongst them were the substitutions S:K417T, S:N440S, S:K444R, S:V445A, S:G446N, S:L452Q, S:N460K, and S:E484V at positions that are notorious for their resistance-mediating effects. The substitution patterns found indicate ongoing adaptive evolution.

## Introduction

While SARS-CoV-2 is usually cleared from the respiratory tract within 10–15 days, occasionally the virus can be detected in the patient for significantly longer, often in conjunction with immunosuppression^1–3^. There have been reports of patients with SARS-CoV-2 infections persisting for up to 500 or even 600 days^4,5^, although these cases have so far been documented on the science news platform EurekAlert! (eurekalert.org) only. The longest infection for which a study has been peer-reviewed and published to date relates to a patient who tested positive for 486 days^6^. Other outstanding cases include patients positive for 471 days^7^, for 335 days^8^, for 245 days^9^, for 242 days^10^, for 236 days^11^, for 218 days^12^, and 216 days^13^, respectively. Some of these cases showed increased amino acid substitution rates^12^, monoclonal antibody escape mutations^6^, or resistance mutations against antivirals such as nirmatrelvir, sotrovimab, and remdesivir^13^. Consequently, viruses from chronically infected patients have the potential to evolve into variants of concern with multiple immune-evading traits and hence should be of high priority in the monitoring of the ongoing evolution of SARS-CoV-2^14^.

Patients with allogeneic hematopoietic stem cell transplantation (HSCT) are known to respond particularly poorly to SARS-CoV-2 vaccination^15^, they clear the SARS-CoV-2 infection more slowly^16^, and have an increased mortality rate in the course of infection^17^. Thus, HSCT recipients are an especially vulnerable group that requires close attention and forms a cohort that offers the virus particularly good conditions for evolutionary adaptations to the human immune system.

Here we present a detailed analysis of the development of the virus population of an immunocompromised patient with B-cell lymphoma and subsequent allogeneic HSCT who was infected with SARS-CoV-2 for at least 521 days. He was finally able to clear the infection. We sequenced the patient’s virus population several times during the infection and discussed the amino acid substitutions that were found in the light of known functional effects and the patient’s medication.

## Methods

### Patient, patient monitoring, testing, and sample acquisition

This study was approved by the local ethics committee (Landesärztekammer Rheinland-Pfalz; Medical Association of the state of Rhineland-Palatinate, Germany) and was conducted in accordance with all relevant ethical guidelines, including the Declaration of Helsinki. The patient provided written informed consent for treatment and publication of his case.

The male patient was diagnosed with stage IV B follicular B-cell lymphoma grade 3a in 2009. He underwent several rounds of chemotherapy and B-cell-directed therapy without achieving a deep remission. In March 2021, an allogeneic HSCT from an HLA-identical donor was performed. The patient developed relapsing autoimmune-mediated hemolytic anemia with non-specific heat antibodies and was treated with B-cell-depleting antibodies and a regimen of the steroid prednisolone initially ranging from 2.5 mg to 5 mg. Acute relapses requiring dose escalation of prednisolone occurred on 23 June 2023 (50 mg, with gradual tapering to 7.5 mg by January 2024) and 19 February 2024 (20 mg, subsequently reduced to 12.5 mg by March 2024). During the entire course after the stem cell transplantation, the patient never recovered a fully functional immune system with persistent quantitative B-cell and T-cell (CD4+ and CD8+) deficiencies. Symptoms associated with COVID-19 included ongoing dyspnea and chronic cough, but not fever, loss of taste, etc.

The patient already had been vaccinated three times with BNT162b2 (BioNTech/Pfizer^18^) against SARS-CoV-2 before the infection, starting from July 2021 onwards, following the recommendations by the Standing Committee on Vaccination at the Robert Koch Institute, Germany, and the German Society for Hematology and Medical Oncology. This scheme is regarded as a primary vaccination, as allogeneic HSCT causes complete B- and T-cell loss. The patient was closely monitored using rapid SARS-CoV-2 antigen tests, and after the first positive test, every one to four weeks by quantitative reverse-transcriptase polymerase chain reaction (q-RT-PCR). This allowed the definition of the infection time with good certainty. The q-RT-PCR tests were performed on GeneXpert GXIV-3-L (Cepheid, Sunnyvale, CA, USA) and NeuMoDx 288 Molecular System (Qiagen, Hilden, Germany) devices. During the time of infection, five patient samples were chosen for sequencing, based on time point and q-RT-PCR threshold cycle (Ct) value.

### Genome sequencing

Virus samples for genome sequencing of SARS-CoV-2 were taken from nasopharyngeal swabs of the routine q-RT-PCR testing of the patient. RNA was isolated on an eMAG platform (bioMérieux, Marcy l’Étoile, France). Viral genomes were amplified using the NEBNext SARS-CoV-2 Sequencing Kit (New England Biolabs, Ipswich, MA, USA) and the Illumina COVIDSeq Test (Illumina, Inc., San Diego, CA, USA), respectively, based on the ARTIC V3 primer set^19^. Sequencing was performed at the Sequencing Consortium’s laboratories^20^ or at StarSEQ GmbH (Mainz, Germany) on Illumina instruments MiSeq, NextSeq 500, and NextSeq 2000 according to standard procedures.

### Sequence data processing

Quality processed sequence reads were mapped to the SARS-CoV-2 reference genome Wuhan-Hu-1 (accession number NC_045512.2). The mean depth across the whole genome (coverage of 99.91%) and the five samples was 9909 sequence reads. A mapping depth of at least 20 was used for the generation of consensus sequences (positions with lower coverage were masked by “N”). All variant sites with less than 90% support for a specific nucleotide were displayed by respective IUPAC nomenclature characters. Generated consensus sequences were then assigned to respective Pango lineages, using the tools pangolin, SCORPIO, and PUSHER and weekly updated pangolin databases^21,22^, following the nCoV-minipipe/CovPipe guidelines^23^.

### Analysis of substitutions and deletions

SARS-CoV-2 genome sequences were aligned in MEGA-X V5 v10.1.8^24^ applying the progressive ClustalW algorithm^25^. Alignments were manually curated and all variant sites were manually inspected. The detected nucleotide substitutions were then examined for their effect on the resulting amino acid sequence using the Gensplore SARS-CoV-2 genome browser^26^. All non-synonymous substitutions that were present in at least two consecutive samples were scored according to the type of amino acid substitution present (conservative or radical, with regard to the properties of the amino acids’ sidechains), the time frame in which the substitution occurred, and the protein encoded by the region in which it was located.

To analyze the location of the amino acid substitutions in the spike protein structure, we downloaded 3D models from the Protein Data Bank^27^ (PDB) and processed them in PyMOL v2.6.0a0 (Schrödinger, New York, NY). We used models of both the spike protein (PDB accession number 6VXX^28^) and the receptor binding domain of the spike protein in binding to the ACE2 receptor (PDB accession number 6LZG^29^).

Genomic deletions that appeared from one-time point to the other and were present in at least two consecutive samples were additionally verified in the respective mappings using a local installation of the Integrative Genomics Viewer v2.17^30^.

The alignment was further used to generate a phylogenetic tree applying the Maximum Likelihood method with the Tamura-Nei model^31^.

## Results

### Patient monitoring, testing, treatment, and sample acquisition

The patient was confirmed to have been SARS-CoV-2-positive for at least 521 days (first positive test on 10/24/2022, last positive test on 03/28/2024), with several negative tests before (last negative test on 08/12/2022) and after this period (first negative test on 04/11/2024), which were carried out as routine tests, as well as to confirm the findings and to detect a resurgence of a possibly only strongly suppressed infection in the case of negative tests.

Treatment was initiated immediately after the first positive PCR test with a single dose of tixagevimab 300 mg and cilgavimab 300 mg (Evusheld®, AstraZeneca AG) and molnupiravir 800 mg twice daily for five days (Lagevrio®, MSD Merck Sharp & Dome AG).

**Fig. 1:**
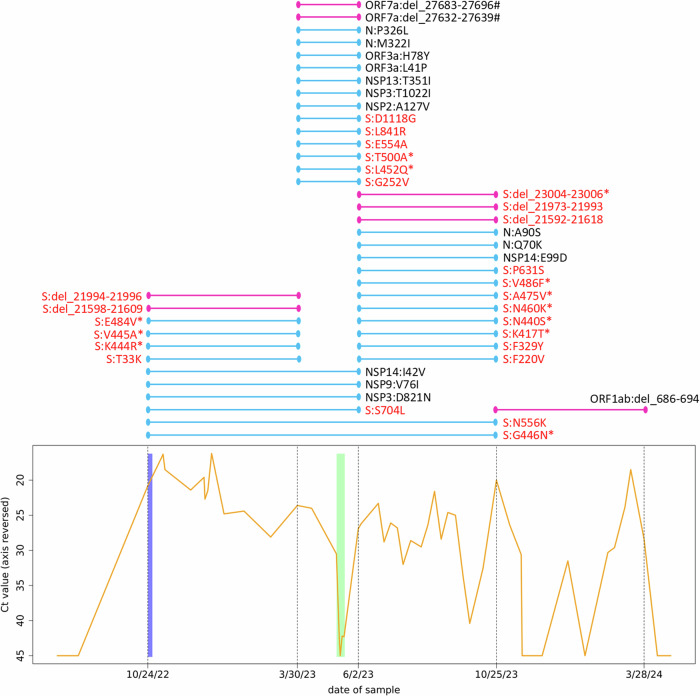
History of the infection and resulting amino acid substitutions and deletions. The graph at the bottom shows the course of the SARS-CoV-2 Ct values of the patient. Note the reversed *y*-axis. Negative tests are displayed as 45 (maximum of the applied test). Additional negative test results at the beginning and end of the shown time slot are documented but not shown. The blue bar behind the curve indicates the period during which the patient was administered tixagevimab and cilgavimab (10/24/2022) as well as molnupiravir (10/24–10/29/2022) as prescribed. The green bar behind the curve indicates the period during which the patient was administered remdesivir as prescribed (05/10–05/19/2023). Dashed vertical lines indicate the time points at which the patient’s virus population was whole-genome-sequenced, with the respective dates on the *x*-axis. The colored lines above this plot show the time windows (between two sequencing events) in which substitutions and deletions have become established in the virus population, each line representing one substitution/deletion. Light blue lines indicate amino acid substitutions (*N* = 34), and dark pink lines indicate deletions (*N* = 8). Deletions are given with genomic nucleotide coordinates. Hash signs after deletions indicate frameshift mutations, resulting in a presumably dysfunctional protein. Lines spanning two or more time intervals show the fixation process of the mutation in the virus population. Substitutions/deletions in the spike protein are highlighted in red, asterisks mark mutations in the receptor binding domain of the spike protein. S spike protein, N nucleocapsid protein, NSP nonstructural protein, ORF open reading frame, Ct value threshold cycle value in q-RT-PCR.

During the infection, the viral load, measured as the Ct value of the q-RT-PCR, fluctuated frequently and significantly (Fig. 1). Because of the ongoing infection, a second treatment was initiated in May 2023 with remdesivir for 10 days (Veklury®, Gilead Sciences). The patient’s most recent SARS-CoV-2 genome sequence (sampled on 3/30/2023) was screened for known remdesivir resistance mutations prior to the treatment.

The patient tested negative several times, once shortly after the remdesivir treatment, but the infection recurred to high viral loads within days or weeks.

### Genome sequencing, variant analysis, and evolutionary rate

Genomes were sequenced to a median depth of 2026 × (standard deviation ± 569). Based on the individual substitution patterns and the application of the pangolin pipeline, all virus samples were scored to belong to the BF.7.21 variant, which is an Omicron BA.5 sub-lineage. All sequences showed the silent C28603T mutation, which is defining for BF.7.21.

**Fig. 2:**
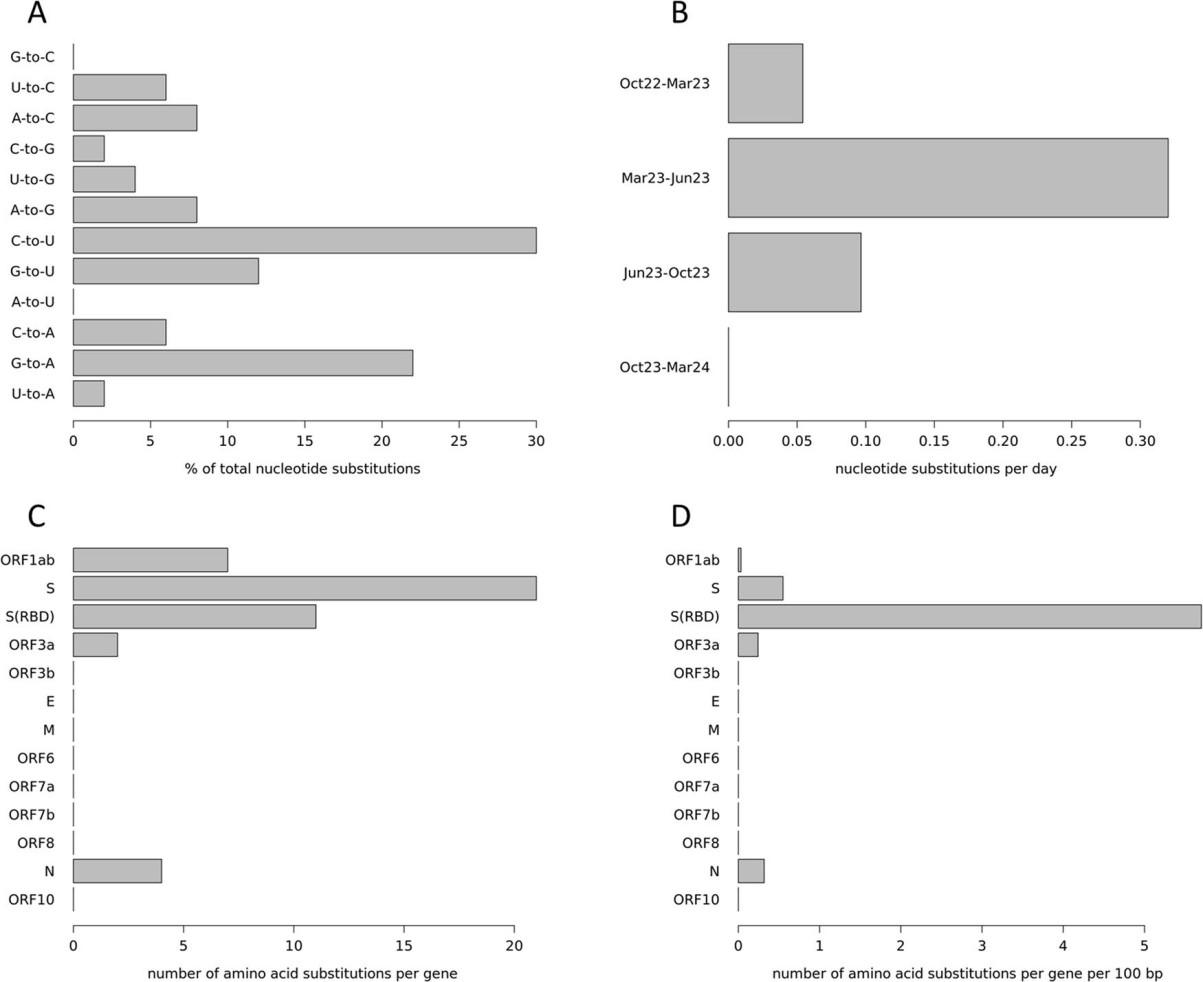
Nucleotide and amino acid substitution patterns. **A** Nucleotide substitutions are detailed by their type, given in percent. For example, “G-to-C” means that a guanine was substituted by a cytosine. **B** Nucleotide substitutions over time. Shown are the periods between two sequencing events, substitution numbers are normalized to substitutions per day. Month names (Mar March, Jun June, and Oct October) are followed by the abbreviated year. **C** Amino acid substitutions per gene. **D** Amino acid substitutions per gene normalized by the gene length. S(RBD) receptor binding domain of the spike protein.

Variant calling revealed 56 sites with a genotype that differed from the reference sequence and that was stable throughout the study period (i.e., the same in all five sequences from the patient’s samples). Moreover, 50 nucleotide positions showed varying genotypes between the five samples (i.e., change of nucleotide within the study period), resulting in an evolutionary rate of 2.87 substitutions per month. The ratio of transversions (pyrimidine against purine or vice versa) to transitions (pyrimidine against pyrimidine, or purine against purine) in the nucleotide substitutions was 32% to 68%, with a clear overrepresentation of substitutions from cytosine to uracil and guanine to adenine (Fig. 2A). The nucleotide substitutions occurred unevenly over the time periods, as defined by the five sequencing events. Many occurred between October 2022 and March 2023 as well as between June 2023 and October 2023 (Fig. 1). However, a markedly higher number of them occurred between March 2023 and June 2023, especially when normalized to the lengths of the time periods (Fig. 2B), and none occurred between October 2023 and March 2024.

Of the 50 nucleotide substitutions found, 34 (68%) resulted in amino acid replacements from early to late samples. The assignment of those amino acid substitutions to the regions coding for the different viral proteins (Fig. 1) showed a strikingly uneven distribution across genes (Fig. 2C), which becomes even more pronounced when normalized to the respective length of the genes (Fig. 2D). Twenty-one (61.8%) amino acid substitutions were found in the region coding for the spike protein which accounts for 12.8% of the genome, corresponding to a 4.8-fold enrichment. Moreover, 11 (32.4%) of these substitutions were within the region encoding for the receptor binding domain (RBD) of the spike protein (which accounts for 2.2% of the genome), resulting in an enrichment of 14.7-fold.

**Fig. 3:**
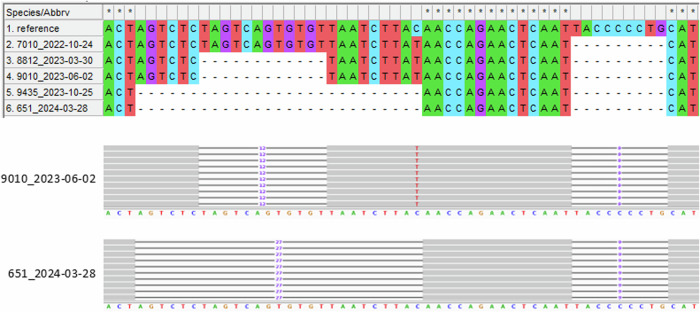
Acquired deletion in the spike protein sequence. The upper part shows the genome region from position 21,589–21,644 for the five samples plus the reference sequence Wuhan-Hu-1 in a MEGA-X alignment. Samples are sorted by sampling date. The prominent, large deletion to the left (12, respectively 27 bp) was acquired in the patient’s virus population in a two-step process: the sequence coding for four amino acids (S:S13, S:Q14, S:C15, and S:V16) was deleted between October 2022 and March 2023, and the coding sequence for another five amino acids (S:V11, S:S12, S:N17, S:L18, and S:T19) was deleted between June 2023 and October 2023. No frameshift or amino acid substitution resulted from the deletions. The corresponding section from the sequence read mapping analysis of one representative sample for both states (short and long deletion) is shown in the IGV screenshot below. The smaller deletion to the right (9 bp) was present in the patient’s virus population from the beginning and resulted in the deletion of three amino acids (S:L24, S:P25, and S:P26) and an amino acid substitution (S:A27S) at the cleavage site.

In addition to these nucleotide substitutions, eight deletions between 9 bp and 27 bp in length were detected in the samples, which newly arose during the study period. Among these, 5 (62.5%) were located in the region encoding for the spike protein (one in its RBD), which corresponds to an enrichment of 4.9-fold (Fig. 1). The deletions were very distinct in their mapping patterns with clear borders and not even single sequence reads extending into the gap (Fig. 3). Two regions from the spike protein showed a very interesting pattern: an initial deletion of several amino acids between October 2024 and March 2023 was followed by an additional deletion in the same spot (thus extending the previous deletion site) between June 2023 and October 2023 (Figs. 1 and 3). Two deletions in ORF7a that occurred between March and June 2023 resulted in frameshifts, presumably rendering the corresponding accessory protein dysfunctional.

**Fig. 4:**
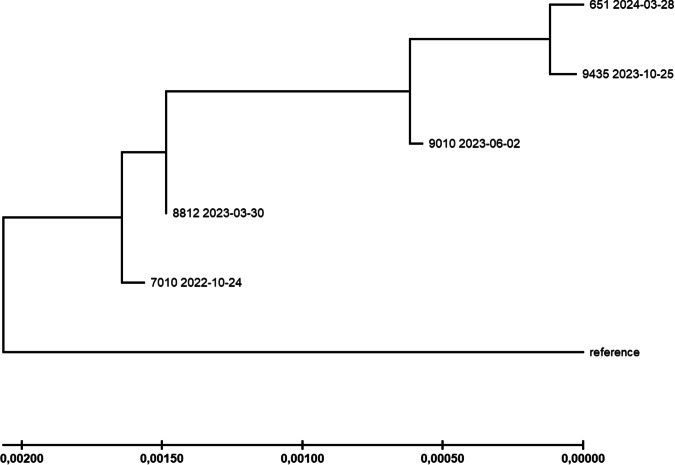
Maximum Likelihood tree. Phylogenetic relationships between the patient’s samples were inferred by the maximum Likelihood method and the Tamura-Nei model. The tree with the highest log likelihood (−41647.52) is shown. The tree is drawn to scale, with branch lengths indicating the number of substitutions per site (see scale at the bottom). Reference = SARS-CoV-2 reference sequence Wuhan-Hu-1. Tree generation was performed with MEGA-X.

The relationships between the individual genome samples were further studied by calculating a phylogeny, and applying the Maximum Likelihood method (Fig. 4). Most importantly, the topology of the resulting tree supported the chronological succession of samples, supporting a stepwise evolutionary trajectory. Moreover, the differences in evolutionary rate with relatively few substitutions between October 2022 and March 2023 and most substitutions between March 2023 and June 2023 were reflected by differing branch lengths as well.

### Structural analysis of amino acid substitutions

**Fig. 5:**
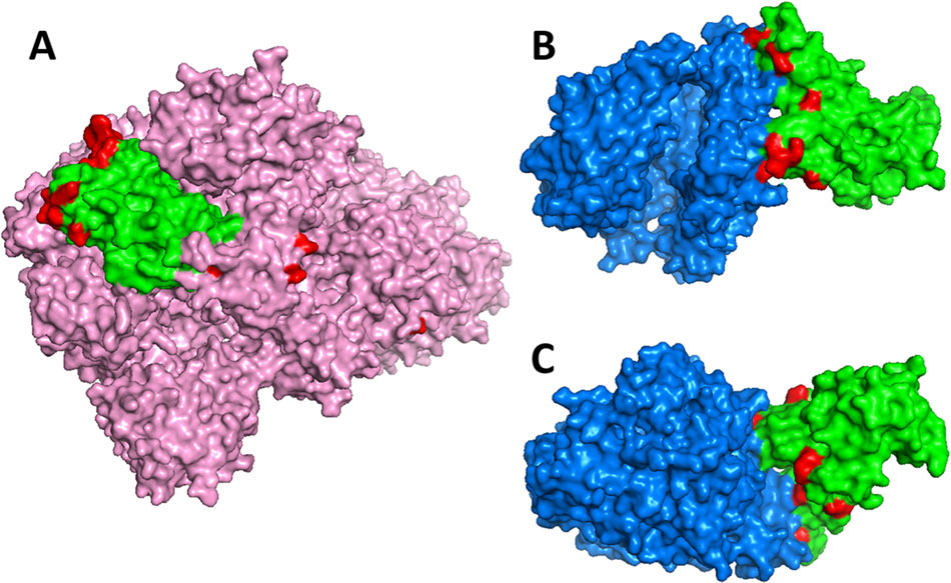
Location of amino acid substitutions in the spike protein. **A** Three-dimensional model of the spike protein (pink) with its receptor binding domain (RBD, green) and substitutions highlighted (red). **B**, **C** Three-dimensional model of the RBD bound to the ACE2 receptor (blue) depicted from two sides, with substitutions highlighted. Protein models visualized in PyMOL.

We found a clear preponderance of amino acid substitutions within the spike protein and its RBD that accumulated in the patient’s virus population during the study period. To study the functional implications of these mutations, we investigated their location by mapping them to a 3D model of the SARS-CoV-2 spike protein in PyMOL (Fig. 5A). As already described, a particularly large number of the substitutions were localized in the spike RBD, where they preferentially accumulated on the outermost surface. To check whether this area has particularly close contact with the target receptor during infection, we also mapped the substitutions onto a 3D model of the spike RBD bound to the human ACE2 receptor (Fig. 5B, C). As suspected, a striking number of substitutions could be found in the immediate contact area between the RBD and the receptor, which makes direct involvement of these mutations in the binding characteristics very likely.

## Discussion

The case of a prolonged SARS-CoV-2 infection described in this study is remarkable for several reasons. First, the infection persisted for an extraordinary duration of 521 days. Additionally, a high number of amino acid substitutions as well as several deletions were observed, with many accumulating in the spike protein and its RBD. Notably, the patient ultimately cleared the virus, allowing the entire period of infection to be monitored from start to finish, including all mutational changes within the patient’s virus population.

Analyzing the mutational patterns of intra-host viral evolution in our case, nucleotide substitutions displayed slightly more transversions than usual for SARS-CoV-2. While the general ratio of transversions to transitions is 0.27 (21% to 79%)^32^, the substitutions found in our patient’s virus population had a transversions to transitions ratio of 0.47 (32% to 68%). Since, for biochemical reasons, the transition mutation rate is inherently higher than the transversion mutation rate^33^, an increased transversion substitution rate hints at a non-random fixation rate that might be driven by adaptive positive selection. When comparing the found distribution of nucleotide substitution types (Fig. 2A) to the mutation spectrum of SARS-CoV-2 at 4-fold degenerate codon sites (which indicates the patterns of evolution at neutrality)^34^, it is noticeable that the virus population in our patient has accumulated an excess of guanine to adenine, cytosine to adenine, and adenine to cytosine substitutions, at the expense of fewer uracil to cytosine and cytosine to uracil substitutions. This shift also indicates the effect of non-neutral mechanisms on the fixation of emerging mutations and thus probably the action of ongoing selection. The most common type of substitution, cytosine to uracil, could indicate host APOBEC-like activity, as proteins from this family have been shown to specifically promote these substitutions in SARS-CoV-2^35,36^ and hence promote virus evolution^37^. This may indicate that the sequence evolution of the patient’s virus population was shaped by two interlocking processes of the patient’s immune system, in the sense that the patient’s APOBEC-like proteins likely increased the mutation rate in the virus’ genome (non-uniformly), and the patient’s antibodies exerted selective pressure on the resulting viral diversity to fix the alleles with the highest adaptive value for evading these specific antibodies.

The rate of amino acid substitutions, which is generally increased in virus populations in immunocompromised patients^38^, was 2.9 amino acid substitutions per month for our case, which is in line with the rates reported from other prolonged SARS-CoV-2 infections in immunocompromised patients (e.g., 1.5–3.8^39^; 2.5^8^; 1.7^9^; and 3.3^12^), resulting in an extraordinarily high number of amino acid substitutions in absolute numbers due to the duration of the infection. Notably, there was an excess of biochemically radical (58.8%) over conservative (41.2%) amino acid substitutions, which contrasts with global patterns in SARS-CoV-2^40^. The radical substitutions are likely associated with changes in the functional properties of the protein, which could be at least partially adaptive. A particularly high number of substitutions was observed in the period between March 2023 and June 2023, visible both at the nucleotide (Figs. 2B and 4) and the amino acid level (Fig. 1). Most interestingly, this period with a particularly high number of substitutions is the shortest between two sequencing runs, and also the period including the administration of remdesivir. However, in contrast to another published case^41^, no remdesivir resistance-mediating mutations were found, suggesting a mechanism other than adaptation to this drug. As a possible alternative scenario explaining the mutational burst, the patient’s virus population was severely decimated by the treatment (as can be clearly seen in the Ct value plot, Fig. 1) and thus went through a genetic bottleneck^42^, which would accelerate fixation of rare alleles and favor high rates of genome change primarily through neutral genetic drift. The high fraction of non-spike protein substitutions in this period (60%) compared to the remaining time (26%) makes drift more likely as the responsible process, illustrating the action of Muller’s ratchet^43^. Alternatively, the non-spike mutations might have been fixed by genetic hitchhiking^44^ with the potentially adaptive spike mutations. The latter consideration could be supported by the fact that the mutations N:M322I and N:P326L, only a few positions apart, were fixed together in this time window. However, the selective value (or neutrality) of the non-spike substitutions introduced into the virus population during this period is currently unclear and subject to further studies. Virus clearance and immediate re-infection can be ruled out, as no more BF.7 lineages were circulating in the region after February 2023, and there was a high degree of overall sequence identity between the samples over time.

Overall, 61.8% of the non-synonymous single nucleotide substitutions (i.e., those that led to amino acid replacements) and 62.5% of the deletions occurred in the genomic region encoding the spike protein, which makes up only 12.8% of the SARS-CoV-2 genome. This is a striking enrichment when compared to typical patterns of substitutions across genes in SARS-CoV-2 genomes that usually show high absolute substitution numbers, especially in ORF1a/b, and high length-normalized rates in ORF3a, the nucleocapsid protein, and ORF10^45^. Even more intriguing in our case, 32.4% of the single nucleotide substitutions that led to amino acid replacements and 12.5% of the deletions occurred in the genomic region encoding the receptor binding domain (RBD) of the spike protein, which makes up only 2.2% of the SARS-CoV-2 genome. These numbers correspond to an enrichment of substitutions and deletions in this specific region, which is responsible for the successful infection of host cells, by up to ~15-fold compared to a random distribution. While one might assume that such a functionally important region is subject to strong purifying selection to ensure its proper function, the RBD has been shown to be considerably mutation tolerant concerning key features like ACE2 affinity and proper folding^46^, providing room for evolutionary experiments. In fact, several of the substitutions found in the virus population of our patient, especially those within the RBD-encoding region, were at positions that are well known for adaptive changes of the spike protein in variants of SARS-CoV-2 that circulated during the pandemic, and three of them (S:N460K, S:L452Q, and S:K444R) were even shown before to be overrepresented in chronic COVID-19 patients^47^. Most of them, however, showed genotypes that differed from the ones most widely distributed worldwide (Table 1). Thus, the changes observed in our patient may represent independent “solutions” that improve viral survival convergently. For illustration, intra-host evolution generated a change at spike protein position K417, with the lysine (K) being replaced by a threonine (T). According to the GISAID database^48^, roughly half of all viruses sequenced to date show an amino acid replacement at this site. However, most of them (98%) show S:K417N, which is known for affecting receptor binding, viral entry, and immune evasion^49,50^. The S:K417T substitution found in our study is only present in 1% of the published sequences (mainly in the Gamma variant) and no functional effects are known. However, the reference amino acid lysine is basic, while the replacement amino acids threonine and asparagine (N) are both polar. Thus, their effects could be similar, although probably not the same. In any case, it is highly suspicious that this well-known and functionally important position was mutated and fixed in the present case. Similar evolutionary patterns showing intra-host changes at sites of population-wide viral divergence can be seen in several other positions, namely S:N440S, S:K444R, S:V445A, S:G446N, S:L452Q, and S:E484V (Table 1). A notable exception is substitution S:N460K. It was found in about two million viral genomes, making up 99.7% of all substitutions at this site. The replacement has been shown to confer pronounced antibody resistance^51–53^, which contributes to the increased infectiousness of more recent SARS-CoV-2 variants such as XBB.1.5, BQ.1.1, or JN.1. Since BF.7.21 of our patient derives from the BA.5 lineage, S:N460K may also indicate a case of convergent evolution, leading to a well understood benefit for the virus.

**Table 1:** Amino acid substitutions that were acquired and fixed in the patient

Nucleotide substitution	Resulting amino acid substitution	Incidence of the substitution	Information on functional effects known of substitutions at this site
C1185T	NSP2:A127V (conservative)	5651 (6760)	–
G5180A	NSP3:D821N (acidic > polar)	6376 (7438)	–
C5784T	NSP3:T1022I (polar > nonpolar)	28,866 (29,650)	–
G12911A	NSP9:V76I (conservative)	4164 (4739)	–
C17288T	NSP13:T351I (polar > nonpolar)	15,039 (15,637)	–
G18163A	NSP14:I42V (conservative)	8,772,722 (8,775,432)	This common substitution seems to be functionally neutral^60^
A18336C	NSP14:E99D (conservative)	283 (959)	–
C21660A	S:T33K (polar > basic)	460 (12,966)	–
T22220G	S:F220V (conservative)	368 (2060)	–
G22317T	S:G252V (conservative)	844,467 (858,813)	–
T22548A	S:F329Y (nonpolar > polar)	26 (1042)	–
A22812C	S:K417T* (basic > polar)	161,674 (8,035,649)	This position is crucial for ACE2 binding by forming a salt bridge with D30 of the receptor^61^; mutations at this position affect receptor binding, viral entry, and immune evasion^49,50^
A22881G	S:N440S* (conservative)	1106 (7,773,564)	Mutations at this position are well known to affect resistance to antibodies and ACE2 binding affinity^62^
A22893G	S:K444R* (conservative)	28,100 (616,810)	Mutations at this position are associated with antibody resistance^62,63^; the specific mutation K444R has been shown to reduce the neutralizing activity of vaccinated sera^64^
T22896C	S:V445A* (conservative)	22,867 (1,261,166)	Mutations at this position have been linked to resistance to neutralization by antibodies^62^; the specific mutation V445A has been shown to reduce the potency of antibodies^65^ and has emerged in a patient with persistent infection^66^
G22898A and G22899A	S:G446N* (nonpolar > polar)	576 (3,339,669)	Mutations at this position are associated with immune evasion functions^67^
G22917A	S:L452Q* (nonpolar > polar)	292,750 (7,837,068)	Mutations at this position are known to affect cellular immunity and infectivity^68,69^; the specific mutation L452Q increases binding to ACE2, evasion of HLA-A24-restricted cellular immunity, and resistance to vaccine-induced antisera^69,70^
T22942G	S:N460K* (polar > basic)	1,961,630 (1,967,965)	This mutation confers antibody resistance^51–53^
C22986T	S:A475V* (conservative)	28,197 (31,410)	This mutation confers resistance to several neutralizing antibodies^71,72^
C23013T	S:E484V* (acidic > nonpolar)	5450 (8,923,843)	Mutations at this position have been associated with neutralization escape and increased viral replicative fitness^73,74^; the specific mutation E484V is relatively rare, but has been found as newly emerged in patients with persistent infections^66^; in addition to the beneficial effects, E484V may be deleterious for ACE2 binding or RBD expression^74^
G23018T	S:V486F* (conservative)	0 (3,976,095)	This is a reverse mutation, from the common F486V mutation back to the wildtype F486; the V486 genotype is associated with immune evasion but also decreased binding affinity to ACE2^75^, hence, the reverse mutation might increase binding affinity to ACE2
A23060G	S:T500A* (polar > nonpolar)	464 (2108)	This position forms a hydrogen bond with Y41 of the receptor^76^; the specific mutation T500A supposedly disrupts antibody binding^77,78^
A23223C	S:E554A (acidic > nonpolar)	1201 (278,344)	Mutations at this position are associated with escape from antibody binding^79^
C23230A	S:N556K (polar > basic)	3874 (5077)	This mutation had no effect in neutralization assays using several monoclonal antibodies^79^
C23453T	S:P631S (nonpolar > polar)	3553 (4372)	–
C23673T	S:S704L (polar > nonpolar)	356,044 (357,465)	This mutation has been shown to decrease infectivity in cell lines^80^
T24084G	S:L841R (nonpolar > basic)	301 (1639)	–
A24915G	S:D1118G (acidic > polar)	312 (1,221,829)	–
T25514C	ORF3a:L41P (conservative)	3424 (41,185)	–
C25624T	ORF3a:H78Y (basic > polar)	280,188 (283,802	This mutation has been associated with higher disease severity^58^
C28481A	N:Q70K (polar > basic)	563 (6861)	–
G28541T	N:A90S (nonpolar > polar)	19,827 (28,472)	–
G29239T	N:M322I (conservative)	9330 (14,100)	–
C29250T	N:P326L (conservative)	6840 (23,814)	Mutations at this position might reduce antibody binding^81^

Genomic positions are relative to the SARS-CoV-2 reference sequence Wuhan-Hu-1. Resulting amino acid substitutions are rated as “conservative” in case the properties of the amino acids’ sidechains do not change with the substitution, or the change in property is indicated. The incidences given are numbers from GISAID and reflect the numbers of sequences published with the very same substitution. The number in parentheses gives the total number of sequences with any kind of substitution at this position. The date of incidence retrieval was 05/28/2024, the total number of GISAID sequences on that date was 16,760,654. An empty field “functional effects” does not mean there are no functional effects of mutations at the site, it rather means there is nothing known about the functional effects of mutations at the site to date.

Asterisks mark substitutions within the spike protein’s receptor-binding domain.

*S* spike protein, *N* nucleocapsid protein, *NSP* nonstructural protein, *ORF* open reading frame.

A particular sequence change that deserves special attention is the substitution of two neighboring amino acids in the RBD region. Both, K444R^54,55^ and V445A^56,57^, have been shown individually to confer resistance to cilgavimab and have already been described as newly emerged in patients treated with this monoclonal antibody. Since both substitutions occurred in the time window right after tixagevimab-cilgavimab administration also in our case, it is plausible to assume that their emergence was driven by the medication. Beyond that, however, there were no signs of monoclonal antibody-driven substitutions.

The only amino acid substitution in our patient’s virus population outside of the spike protein coding region for which we found evidence of a functional effect was ORF3a:H78Y, which has been associated with higher disease severity^58^. All deletions in the region encoding the spike protein were multiples of three, so that only some amino acids were removed from the resulting protein, but the reading frame was not disrupted. However, the two deletions in the ORF7a resulted in a frameshift and thus likely a dysfunctional protein. The ORF7a accessory protein plays several roles in the viral life cycle and in the interaction with the host, but is non-essential for viral replication. Its impairment may have reduced the efficiency of the immune evasion of the virus by disabling ORF7a’s tetherin blocking function^59^, a disadvantage that could probably only become established in the context of a weakened immune system.

A limitation of the study is the fact that only nasopharyngeal swabs were used. This may underestimate the diversity of the virus population by missing subpopulations in the deeper airways or even in the gut. Following this line of thought, the interim negative q-RT-PCR results could therefore be due to temporally very low virus load at this specific site only.

We did not isolate the virus from the patient and therefore cannot say anything about the actual infectivity of the virus during different stages of the in-patient evolution. We also did not perform in-depth analyses of the patient´s immune system and function, such as neutralizing antibodies or COVID-19-specific T-cell responses. Therefore, the influence of the patient´s immune system on the evolution of the virus can only be indirectly inferred from the acquisition of mutations. The many substitutions in the RBD of the spike protein are most likely adaptive overall and have probably fine-tuned the binding properties to antibodies on the one hand and to the ACE2 receptor on the other. Whether these were adaptations that were very specifically tailored to the immune system of the patient is difficult to say and would require functional tests. However, many such cases will eventually also produce adaptations that sustainably increase infectivity or other parameters of the virus.

We present here a case of extremely persistent SARS-CoV-2 infection in an immunocompromised patient. During the 521 days of infection, the virus population acquired many amino acid substitutions and several deletions, a strongly overrepresented fraction of which were found in the receptor binding domain of the virus’ spike protein. The substitution patterns, the presumed functions of the respective amino acids, the temporal sequence, and the position of the substitutions in the spike protein are strongly indicative of functional adaptation triggered by selection. No known adaptations to the administered drug remdesivir were found, but signs of adaptation to the administered monoclonal antibodies could be observed. Overall, adaptation of the virus to the immune system seemed to be the main driving force. Such a prolonged course of infection, with constant but weak selection pressure from the immune system combined with accelerated fixation rates due to bottlenecks triggered by drug treatments, actually favors a targeted evolution towards increased virus fitness. This case, together with others published, may help to better understand the evolutionary pathways of SARS-CoV-2 in immunocompromised patients with long-term infections, and ultimately improve medical support and treatment for this highly vulnerable cohort.

## Data Availability

Whole genome re-sequencing data is available at the European Nucleotide Archive (https://www.ebi.ac.uk/ena/browser/home) under the project accession number PRJEB77414.
